# Biodistribution and dosimetry for combined [^177^Lu]Lu-PSMA-I&T/[^225^Ac]Ac-PSMA-I&T therapy using multi-isotope quantitative SPECT imaging

**DOI:** 10.1007/s00259-022-06092-1

**Published:** 2023-01-11

**Authors:** Astrid Delker, Mirjam Schleske, Grigory Liubchenko, Isabella Berg, Mathias Johannes Zacherl, Matthias Brendel, Franz Josef Gildehaus, Mikhail Rumiantcev, Sandra Resch, Kerstin Hürkamp, Vera Wenter, Lena M. Unterrainer, Peter Bartenstein, Sibylle I. Ziegler, Leonie Beyer, Guido Böning

**Affiliations:** 1 Department of Nuclear Medicine, University Hospital, LMU Munich, Munich, Germany; 2 SyNergy, University of Munich, Munich, Germany; 3 DZNE – German Center for Neurodegenerative Diseases, Munich, Germany; 4 Institute of Radiation Medicine, Helmholtz Zentrum München, German Research Center for Environmental Health GmbH, 85764 Neuherberg, Germany

**Keywords:** PSMA, Prostate cancer, SPECT, ^225^Ac, Dosimetry

## Abstract

**Purpose:**

Quantitative SPECT for patient-specific dosimetry is a valuable tool in the scope of radionuclide therapy, although its clinical application for ^225^Ac-based treatments may be limited due to low therapeutic activities. Therefore, the aim of this study was to demonstrate the feasibility of clinical quantitative low-count SPECT imaging during [^177^Lu]Lu-PSMA-I&T/[^225^Ac]Ac-PSMA-I&T treatment.

**Methods:**

Eight prostate cancer patients (1000 MBq/8 MBq [^177^Lu]Lu-PSMA-I&T/[^225^Ac]Ac-PSMA-I&T) received a single-bed quantitative ^177^Lu/^225^Ac SPECT/CT acquisition (1 h) at 24 h post treatment (high-energy collimator, 16 projections p. head à 3.5 min, 128 × 128 pixel). The gamma peak at 440 keV (width: 10%) of the progeny ^213^Bi was imaged along with the peak at 208 keV (width: 15%) of ^177^Lu. Quantification included CT-based attenuation and window-based scatter correction plus resolution modelling. Gaussian post-filtering with a full-width-half-maximum of 30 mm and 40–45 mm was employed to match the signal-to-noise ratio of ^225^Ac and ^177^Lu, respectively.

**Results:**

Kidney (*r* = 0.96, *p* < 0.01) and lesion (*r* = 0.94, *p* < 0.01) SUV for [^177^Lu]Lu-PSMA-I&T and [^225^Ac]Ac-PSMA-I&T showed a strong and significant correlation. Kidney SUV were significantly higher (*p* < 0.01) for [^225^Ac]Ac-PSMA-I&T (2.5 ± 0.8 vs. 2.1 ± 0.9), while for [^177^Lu]Lu-PSMA-I&T lesion SUV were significantly higher (*p* = 0.03; 1.8 ± 1.1 vs. 2.1 ± 1.5). For absorbed dose estimates, significant differences regarding the kidneys remained, while no significant differences for lesion dosimetry were found.

**Conclusion:**

Quantitative low-count SPECT imaging of the peak at 440 keV during [^225^Ac]Ac-PSMA-I&T therapy is feasible. Multi-isotope imaging for [^177^Lu]Lu-PSMA-I&T/[^225^Ac]Ac-PSMA-I&T therapy indicates accumulation of free ^213^Bi in the kidneys.

**Supplementary Information:**

The online version contains supplementary material available at 10.1007/s00259-022-06092-1.

## Introduction

Prostate cancer is top ranked for the worldwide deaths of men [1]. Radioligand therapy using radioactive-labeled ligands that are able to target the prostate-specific membrane antigen (PSMA) evolved as a promising strategy for the diagnosis and treatment of metastasized castration-resistant prostate cancer (mCRPC). Regarding therapy, PSMA ligands like PSMA-617 or PSMA-I&T are currently in use with the β^−^-emitter ^177^Lu or the α-emitter ^225^Ac [2–7]. α-emitters like ^225^Ac are characterized by a high biological effectiveness for cell killing due to the highly localized energy deposition, i.e., a high linear energy transfer (LET). By combining low-LET and high-LET radiation qualities, e.g., by using ^177^Lu and ^225^Ac in tandem, the therapeutic window can be effectively increased. More precisely, the radiation harm to healthy tissues can be reduced, while the probability of irrepairable damage to malignant cells can be increased [8].

Quantitative post-therapy imaging and associated dosimetry can contribute to further increase the patient-specific therapeutic window or to identify mechanisms of general relevance for therapy response. Image-based dosimetry using quantitative single-photon-emission-computed-tomography (SPECT) or positron-emission-tomography (PET) imaging allows to directly assess the patient-specific post-therapeutic radionuclide distribution in vivo and to determine the radiation dose for an organ, a tumor, or even a single voxel [9]. For ^177^Lu-based therapy, quantitative SPECT imaging is an established means [10]. However, for ^225^Ac-based therapy, the low therapeutic activities of several MBq so far limited the integration of quantitative imaging into clinical routine, although as part of the decay of ^225^Ac and its subsequent daughters, two gamma components are emitted with in principle sufficient emission probability for SPECT imaging (218 keV: 11.4%; 440 keV: 25.9%) [11]. The gamma component of 218 keV originates from the decay of the first daughter ^221^Fr and could be imaged in a similar manner as the 208 keV photopeak of ^177^Lu. The gamma component of 440 keV is emitted during the decay of the progeny ^213^Bi and lies above the typical energy range of conventional SPECT imaging [9]. Quantitative SPECT imaging of high-energy photons poses an additional technical challenge, as usually pronounced septal penetration through the collimator is present and special effort has to be taken regarding modelling of the collimator-detector-response (CDR) [12].

Several studies already addressed clinical qualitative imaging for radionuclide therapy using ^213^Bi alone [13, 14]. Sgouros et al. performed image-based dosimetry for ^213^Bi-HuM195 (anti-CD33) therapy of patients with leukemia using planar acquisitions [15]. However, due to the shorter half-life of ^213^Bi and the release of only a single alpha particle compared to the whole ^225^Ac decay chain, the employed therapeutic activities for sole ^213^Bi-based treatments are higher, i.e., in the order of several hundred MBq to GBq. Furthermore, SPECT imaging is of advantage for quantification purposes compared to planar imaging, as there is less overlap of accumulating tissues and advanced methods for correction of image-degrading effects can be directly considered within iterative reconstruction [9]. First attempts for SPECT imaging for ^225^Ac-based treatments already exist for phantom studies using microSPECT/CT acquisitions of both, ^221^Fr and ^213^Bi, or for the clinical setting using a multi-peak reconstruction approach to employ the maximum available signal information [11, 16, 17]. However, in particular the quantification of post-therapeutic imaging for ^225^Ac radionuclide therapy in the scope of individualized dosimetry is still an open issue. The aim of this study is to demonstrate the clinical feasibility of quantitative low-count SPECT imaging for [^225^Ac]Ac-PSMA-I&T therapy of advanced prostate cancer and to provide a first image-based estimate of the radiation absorbed dose in critical organs and lesions. The patient cohort presented in this study received a simultaneous injection of both, [^177^Lu]Lu-PSMA-I&T and [^225^Ac]Ac-PSMA-I&T. This special patient cohort offers the unique opportunity to directly compare both compounds in vivo and to investigate low-count imaging of the 440 keV photopeak of the ^225^Ac-progeny ^213^Bi.

## Material and methods

### Patients and therapy

Eight patients who received both, on average 7.96 ± 0.03 MBq [^225^Ac]Ac-PSMA-I&T and on average 1057 ± 20 MBq [^177^Lu]Lu-PSMA-I&T, were included in this study. More detailed data on the patient characteristics can be found in the supplemental information. [^225^Ac]Ac-PSMA-I&T was injected freehand, while [^177^Lu]Lu-PSMA-I&T was administered via an infusion pump. Further radiopharmaceutical and treatment details are described in [4, 18]. PSMA expression was verified prior to therapy via [^18^F]F-PSMA-1007 PET/CT imaging. After injection, all patients were hospitalized for 48 h in agreement with the German regulations on radiation protection. Approximately 24 h post injection (p. i.), each patient obtained combined single-bed ^177^Lu/^225^Ac SPECT/CT imaging with a total scan time of 1 h. The scan region was selected according to the pre-therapeutic PSMA PET in such a way that both kidneys and as many lesions as possible were included in the field-of-view (FOV). Forty-eight hours post-therapy, an additional 20-min three-bed scan was acquired using the ^177^Lu energy windows alone, to additionally visualize the whole-body uptake pattern, which was in the first place not feasible for the prolonged scan time for combined ^177^Lu/^225^Ac imaging. The scan at 24 h was employed to compare [^225^Ac]Ac-PSMA-I&T and [^177^Lu]Lu-PSMA-I&T uptake patterns, while both ^177^Lu acquisitions were further employed to estimate the patient-specific biological half-life of the PSMA ligand. In the case of long examination times, patient positioning is a crucial aspect of patient comfort. Patients were positioned with arms along the torso and an arm fixation via a cloth belt around the abdomen was employed. Furthermore, patient positioning was adjusted with knee rolls or soft pillows according to the patient’s individual desire. All employed devices were noted in the patient-specific scanning protocol to allow for a comparable positioning at 24 and 48 h.

This study is based on retrospective and irreversibly anonymized patient data and was approved by the local ethics committee (project no. 22–0544). All patients gave written consent to undergo radioligand therapy, which was performed in agreement with the updated Declaration of Helsinki (§37) and the German Medical Products Act (§13.2b).

### Image acquisition

SPECT imaging was performed on a dual-headed Siemens Symbia Intevo T16 SPECT/CT (Siemens Healthineers, Erlangen, Germany) equipped with a high-energy collimator, to minimize artifacts caused by septal penetration of the 440 keV gammas emitted from the daughter nuclide ^213^Bi. Combined ^177^Lu/^225^Ac SPECT/CT imaging at 24 h post-therapy employed the 440 keV photopeak of the ^225^Ac decay chain (width: 20%), similar to previous imaging studies for ^213^Bi [13, 15], and the 208 keV photopeak for ^177^Lu (width: 15%) [10, 18]. The contribution of the 218 keV gamma emission of the ^225^Ac daughter nuclide ^221^Fr to the 208 keV window was neglected, as for an ^225^Ac-to-^177^Lu activity ratio of 8:1000 this contribution is expected to be below 1%. Additional scatter windows were acquired for both aforementioned photopeaks, i.e., a lower adjacent scatter window of 10% width for 440 keV, plus a lower and an upper scatter window at 170 keV (width: 15%) and 240 keV (width: 10%) for the peak at 208 keV. Sixteen projections per head were acquired at a step time of 3.5 min with 128 × 128 pixels (4.7952 × 4.7952 mm^2^). Number of projections and time per angular view were selected in such a way that the total scan time per bed position does not exceed 1 h. In the low-count situation, an increase in step time was preferred over a high number of views. ^177^Lu SPECT/CT imaging at 48 h p. i. employed the energy window settings as specified above and was performed according to the clinical acquisition protocol, i.e., 64 projections per head at 5 s per projection using 128 × 128 pixels (4.7952 × 4.7952 mm^2^). A CT scan for attenuation correction was acquired with a slice thickness of 3 mm, at 110 keV tube voltage, and with CARE Dose enabled using 15 mAs as a reference tube current.

### Image reconstruction

Image reconstruction and quantification were performed via the in-house MAP-EM algorithm described in [18]. This included CT-based attenuation correction and window-based scatter correction, i.e., the triple-energy-window method for ^177^Lu and the dual-energy-window method for ^225^Ac [9, 10]. For ^177^Lu, CDR modelling was achieved via a distance-dependent Gaussian resolution model. By contrast, the high-energy 440 keV gammas of ^225^Ac cause a pronounced anisotropic septal penetration pattern. Thus, modelling of the ^225^Ac CDR employed a stack of 150 2D point-spread-functions (PSF), which were simulated via the SIMIND Monte Carlo code (Version 6.2.1) for distances up to 60 cm [12]. As the number of counts per projection was low during ^225^Ac reconstruction, all views were employed to calculate the image update. By contrast, for ^177^Lu, 16 projections per subset and 20 iterations were used according to the conventional local protocol. To enable absolute image quantification, for each nuclide, a respective calibration factor was determined using a large cylindrical volume homogeneously filled with a known activity concentration as described in [9].

### Phantom measurement: quantification of recovery and signal-to-noise ratio

**Fig. 1:**
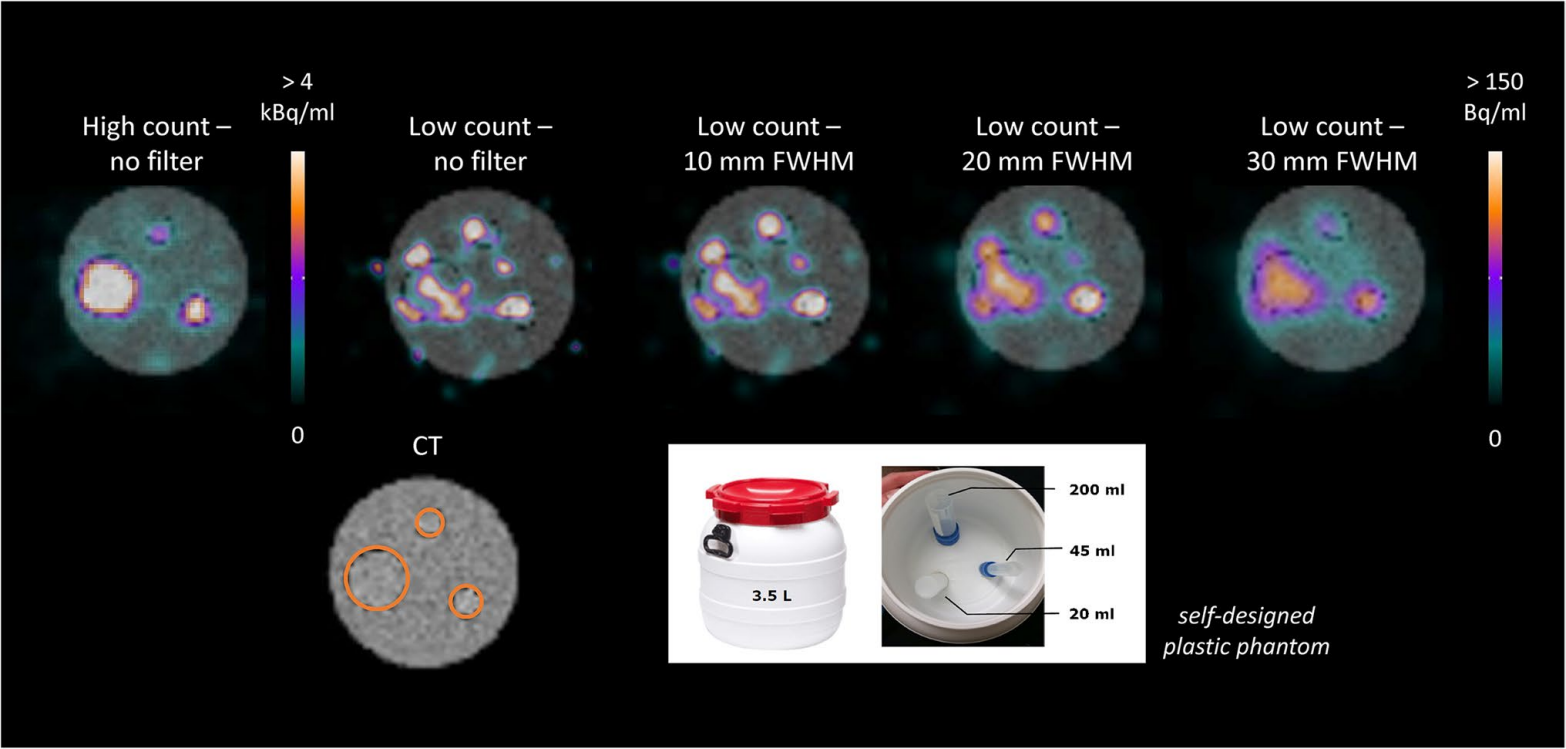
Transversal slice of the self-designed phantom filled with ^225^Ac at a foreground-to-background ratio of 6.4:1; the high-count situation corresponds to a total phantom activity of 4.5 MBq and the low-count situation reflects the clinical case with a 30-fold lower signal strength

To investigate the general feasibility of clinical ^225^Ac SPECT imaging, a self-designed plastic phantom was used (Fig. 1). The phantom was composed of a large cylindrical volume of 3.5 l, and three fillable inserts of approximately 200, 45, and 20 ml. The size of the inserts shall mimic an approximate volume of a kidney and a salivary gland as well as a typical lesion size, which all represent structures of interest for PSMA therapy. Furthermore, the activity concentration of the selected inserts aimed at approximately 200 Bq/ml, according to the expected average activity concentration in critical organs at 72 h post [^225^Ac]Ac-PSMA-I&T therapy and under the assumption of an injected activity of approx. 8 MBq. This activity concentration was determined from the biological half-life of 15 prostate cancer patients receiving [^177^Lu]Lu-PSMA-I&T therapy, assuming equal biological pharmacokinetics for [^177^Lu]Lu-PSMA-I&T and [^225^Ac]Ac-PSMA-I&T as a first approximation. Seventy-two hours usually represent the latest time point to be imaged according to the local institutional procedures for ^177^Lu-based therapy, which might also become the minimum desired late time point for ^225^Ac-based therapy [18]. Within this setting, both, a high-count (HC) and a low-count (LC) measurement, were performed. The LC measurement shall reflect clinical conditions as described above, while the HC measurement shall be used to investigate the general quantification capabilities for 440 keV. Thus, for the HC measurement, an approximate 30-fold increase in ^225^Ac activity was employed according to the maximum available activity at that point, resulting in a total activity of approximately 4.5 MBq. To simulate a clinically realistic signal strength for the LC case, the measurement time per projection was decreased by a factor of 30.

For both, LC and HC measurement, recovery coefficients (RC) for all three inserts were determined by using volumes-of-interest (VOI) drawn on the CT. For the background RC, the average activity concentration in five 100-ml rod-shaped background VOI, placed between and around the inserts, was measured. Furthermore, the signal-to-noise ratio (SNR) was determined for all three inserts. To improve the SNR in particular for the LC situation, three Gaussian post-reconstruction filters with a full-width-half-maximum (FWHM) of 10, 20, and 30 mm were investigated.

Real phantom measurements were accompanied by SIMIND simulations of a computational phantom containing three large spheres of 200, 26.5, and 11.5 ml volume, to compare image quantification capabilities for ^177^Lu and ^225^Ac for a well-known ground truth [19]. Volumes were selected similar to a typical kidney volume and according to the largest spheres of a conventional NEMA IEC body phantom. The simulation parameters related to camera system and acquisition protocol as well as the reconstruction settings were identical to those employed for the real-life measurements. Results from the simulation analysis can be found in the supplemental information.

### Patient-specific biodistribution and dosimetry

In a first step, the biodistribution of both, [^225^Ac]Ac-PSMA-I&T and [^177^Lu]Lu-PSMA-I&T, was assessed based on the multi-isotope SPECT images at 24 h. Therefore, both kidneys were contoured on the CT. Due to both, the different count situation and the different spatial resolution, initial ^177^Lu and ^225^Ac SPECT images were challenging to compare due to apparent differences in particular for the nuclide-specific SNR. This impacted the co-localization of lesions in both images. Thus, Gaussian post-filtering was employed to harmonize ^177^Lu and ^225^Ac image qualities as closely as possible. A FWHM of 45 mm and 30 mm was chosen for ^177^Lu and ^225^Ac, respectively, to harmonize the expected kidney SNR, as well as 40 mm and 30 mm to allow for a comparison of the lesion biodistribution. The respective FWHM was determined based on the SIMIND simulations presented in the supplement. Gaussian post-filters with a varying FWHM (20–50 mm in steps of 5 mm) were investigated to achieve the closest match of the insert SNR. Furthermore, respective Gaussian post-filters were applied to the patient data and an experienced user visually judged similarity of both, [^177^Lu]Lu-PSMA-I&T SPECT and [^225^Ac]Ac-PSMA-I&T SPECT images, using the pre-therapy PSMA PET/CT for orientation. The lesion uptake for [^225^Ac]Ac-PSMA-I&T and [^177^Lu]Lu-PSMA-I&T was finally derived on the post-filtered image data based on an isocontour of 80% of the maximum (PMOD version 3.609), which, according to the simulation study, provided the closest match to the known sphere volume.

Due to the prolonged scan time, ^225^Ac SPECT imaging at this point was only performed at a single time point. However, methods for single-point dosimetry were already intensively investigated for ^177^Lu-PSMA therapy and ^177^Lu-PRRT (peptide receptor radionuclide therapy) [20–25]. Most of the proposed methods employ a population-based effective half-life or use the effective half-life measured for a previous treatment cycle in combination with a single uptake measurement to estimate the absorbed dose for organs-at-risk or tumors. In this study, ^225^Ac image-based dosimetry was performed using the pharmacokinetic information from serial ^177^Lu imaging as performed at 24 h and 48 h post injection. More precisely, the ^177^Lu scan acquired at 48 h p. i. was co-registered to the ^177^Lu SPECT at 24 h (PMOD version 3.609) and kidney and lesion VOI as delineated before on the ^177^Lu SPECT/CT 24 h p. i. were manually adjusted to the scan at 48 h, if necessary. Decay correction was applied to all VOI data to account for the different physical half-lives of ^177^Lu and ^225^Ac, prior to a mono-exponential fit to the data points. To obtain 3D absorbed dose distributions for both nuclides, the VOI-specific effective half-life was applied to each voxel being part of the considered VOI. In particular, the time-integrated activity of each voxel assigned to a specific VOI is given by:

$$TIA({\varvec{x}})= A({\varvec{x}},{t}^{*})\bullet \mathrm{exp}\left(\frac{\mathrm{ln}\left(2\right)}{{T}_{\frac{1}{2}, VOI}}\bullet {t}^{*}\right)\bullet \frac{{T}_{\frac{1}{2},VOI}}{\mathrm{ln}\left(2\right)}$$

$TIA({\varvec{x}})$ denotes the time-integrated activity of a voxel at position ${\varvec{x}}$ within a certain VOI, $A({\varvec{x}},{t}^{*})$ is the activity uptake of this voxel at the reference time point ${t}^{*}$ around 24 h, and ${T}_{\frac{1}{2}, VOI}$ is the VOI-specific effective half-life. Absorbed dose maps were generated via a voxel S-value kernel (VSV) for ^225^Ac and ^177^Lu, which were pre-simulated via the FLUKA Monte Carlo code (version: FLUKA 2021.2.2; © Alberto Fassò, Alfredo Ferrari, their collaborators, and INFN) [26–28]. The VSV was initially derived for soft tissue, whereupon absorbed dose maps were weighted with the local CT density in a voxel-wise manner [29]. To account for the higher biological effectiveness for alpha particles compared to beta radiation, a relative biological effectiveness (RBE) of five as proposed by Sgouros et al. was applied for ^225^Ac, in contrast to a RBE of one for ^177^Lu [30].

Kidney and lesion standardized uptake values (SUV) as well as absorbed doses were evaluated for correlation and significant differences using MATLAB Pearson correlation analysis and MATLAB Wilcoxon signed-rank testing.

## Results

### Phantom measurement

**Fig. 2:**
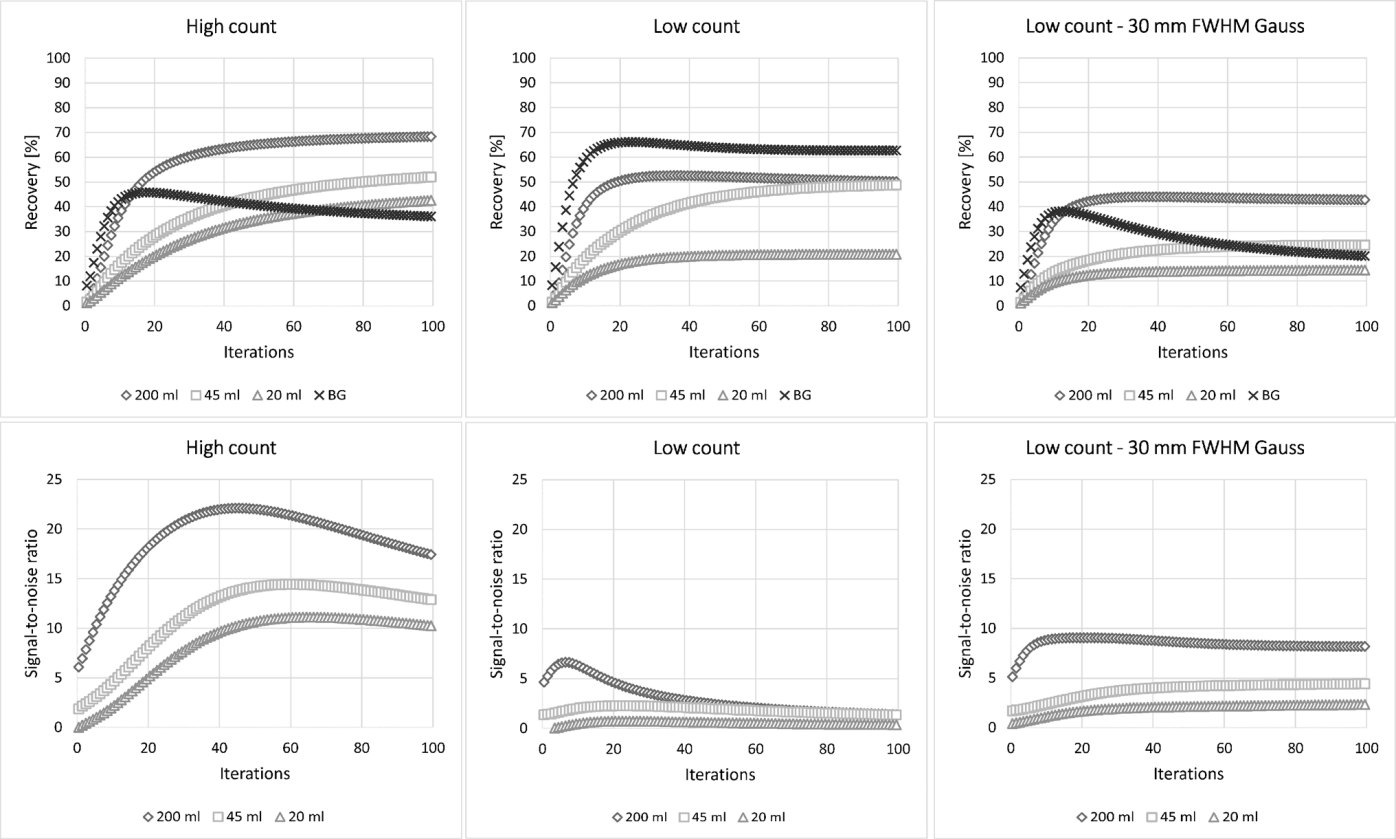
Recovery coefficients and signal-to-noise ratio for the self-designed phantom of Fig. 1 (BG: background)

Figure 1 illustrates a transversal slice of the self-made phantom for both, the high-count (HC) and low-count (LC) condition. The foreground-to-background ratio for the phantom measurement was approximately 6.4:1. For the HC case, all three inserts are clearly visible, well positioned, and accurately shaped. However, for the LC situation, the unfiltered signal is clearly distorted and too noisy to allow for identification of the inserts. Gaussian post-filtering with a FWHM > 20 mm is required for noise suppression and to adequately restore the insert signal. A FWHM of 30 mm lead to a reasonable signal distribution, i.e., signal shape and position. Figure 2 shows the evaluated image RC and SNR in dependence upon the number of MLEM iterations. For the HC situation, a RC of up to 70% and 40% is obtained for the largest and smallest insert, respectively, while a SNR > 10 can be achieved for all inserts. For the LC situation, a pronounced drop of the SNR for all inserts is noticeable. Gaussian post-filtering with a FWHM of 30 mm improves the SNR in particular for the largest insert up to approximately 10, at the cost of a loss of RC. A number of 60 iterations were considered reasonable for the clinical LC situation, as with a higher number of iterations no significant improvement in RC or SNR could be reached. Furthermore, MLEM reconstruction is well known to cause noise amplification with higher number of iterations. It can be noticed especially for Fig. 2 that the background signal is not fully recovered for none of the investigated cases.

The calibration factor for ^225^Ac was found to be 22.7 cps/MBq, opposed to a ^177^Lu calibration factor of 7.0 cps/MBq for the high-energy collimator.

### Patient-specific biodistribution and dosimetry

**Fig. 3:**
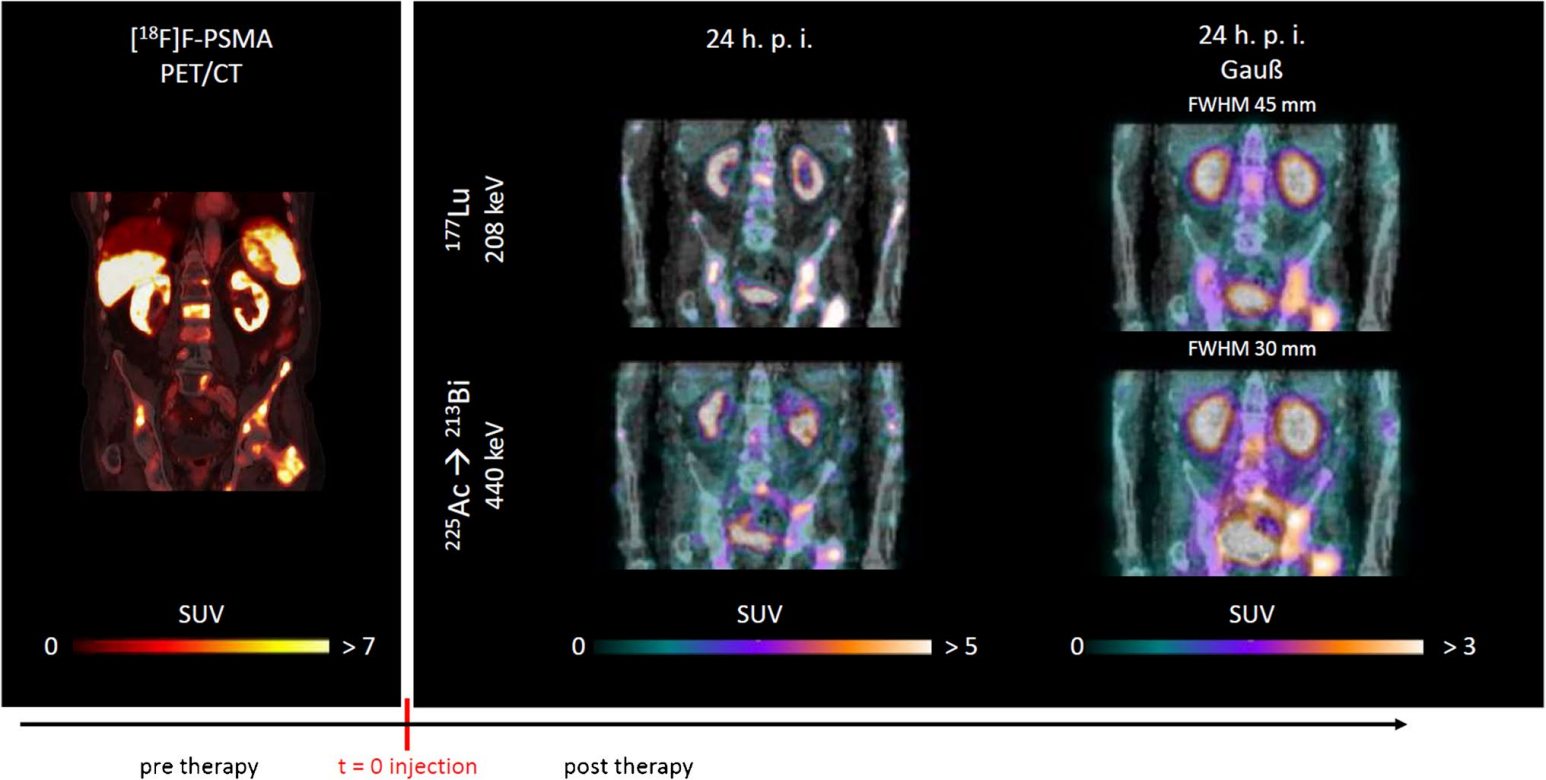
Coronal slices for patient 5; the pre-therapeutic PSMA PET/CT is shown together with the filtered and unfiltered therapeutic quantitative SPECT/CT for [^177^Lu]Lu-PSMA-I&T and [^225^Ac]Ac-PSMA-I&T at 24 h p. i

**Fig. 4:**
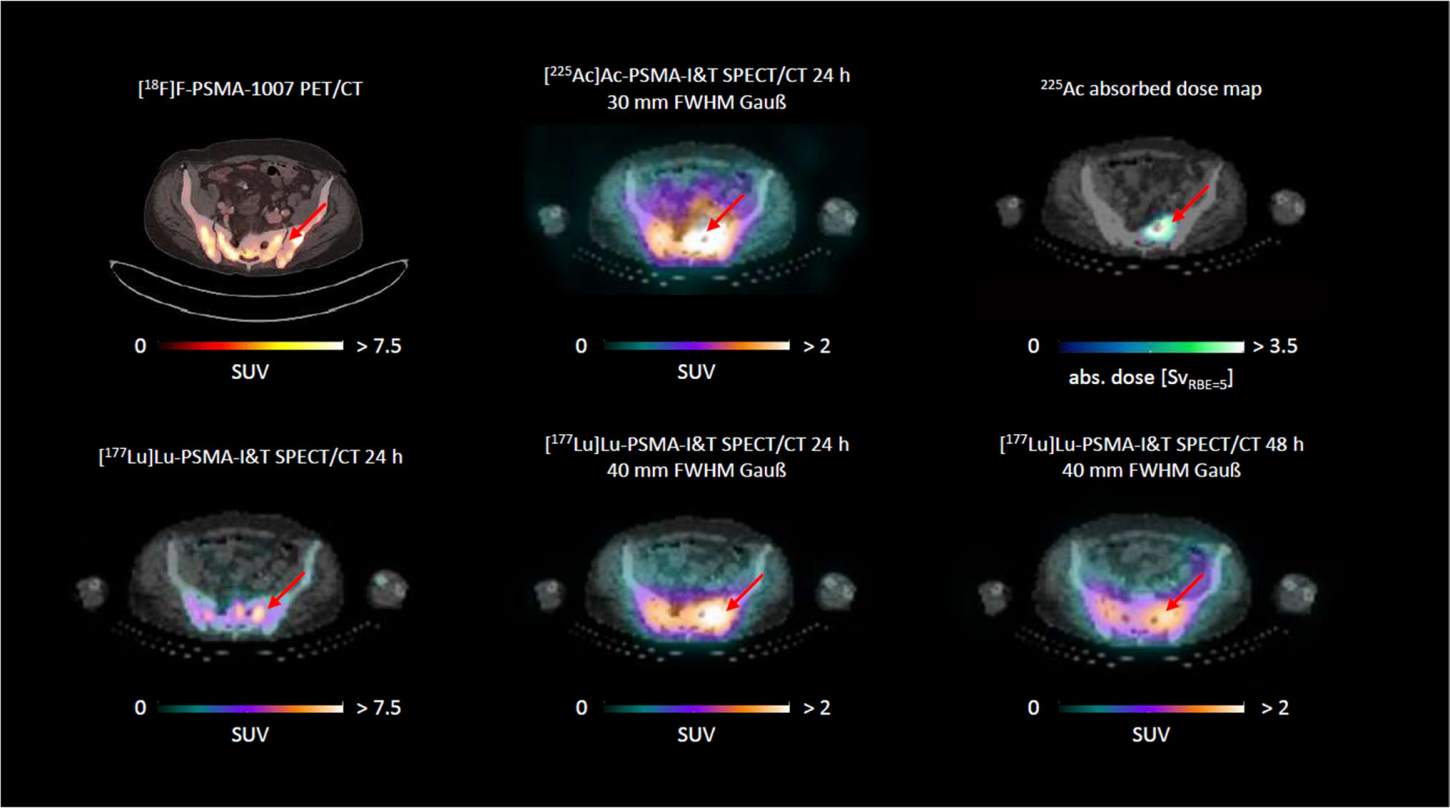
Transversal slices of the pelvic region for patient 5; the pre-therapeutic PSMA PET/CT is shown together with the quantitative [^177^Lu]Lu-PSMA-I&T SPECT/CT at 24 h and 48 h p. i. and the single quantitative [^225^Ac]Ac-PSMA-I&T SPECT/CT at 24 h, as well as the resulting ^225^Ac RBE-weighted absorbed dose distribution

Measured count rates per projection at 24 h p. i. were 0.3 cps/keV for ^225^Ac (26 cps for 440 keV ± 10%) and 10.3 cps/keV for ^177^Lu (321 cps for 208 keV ± 7.5%). Figure 3 illustrates both, the ^177^Lu and ^225^Ac SPECT/CT at 24 h exemplarily for patient 5 with and without Gaussian post-filtering. The pre-therapy PSMA PET/CT is additionally shown for comparison. Gaussian post-filtering leads to a comparable uptake pattern for both, [^177^Lu]Lu-PSMA-I&T and [^225^Ac]Ac-PSMA-I&T, respectively ^213^Bi. Figure 4 additionally shows the pre-therapy PSMA PET/CT, the [^225^Ac]Ac-PSMA-I&T SPECT/CT at 24 h, and the [^177^Lu]Lu-PSMA-I&T SPECT/CT at 24 h and 48 h p. i. for the pelvic region. Due to the limited resolution for ^225^Ac SPECT imaging, individual metastases cannot be resolved as it is the case for PET or unfiltered ^177^Lu SPECT imaging. Nonetheless, the uptake distribution is similar and appears to be reasonable, in particular in comparison to the harmonized ^177^Lu SPECT. In addition, the RBE-weighted absorbed dose distribution (Sv_RBE=5_) is shown for the high-uptake region in the right pelvis. Figures for more patient cases can be found in the supplemental information.

**Fig. 5:**
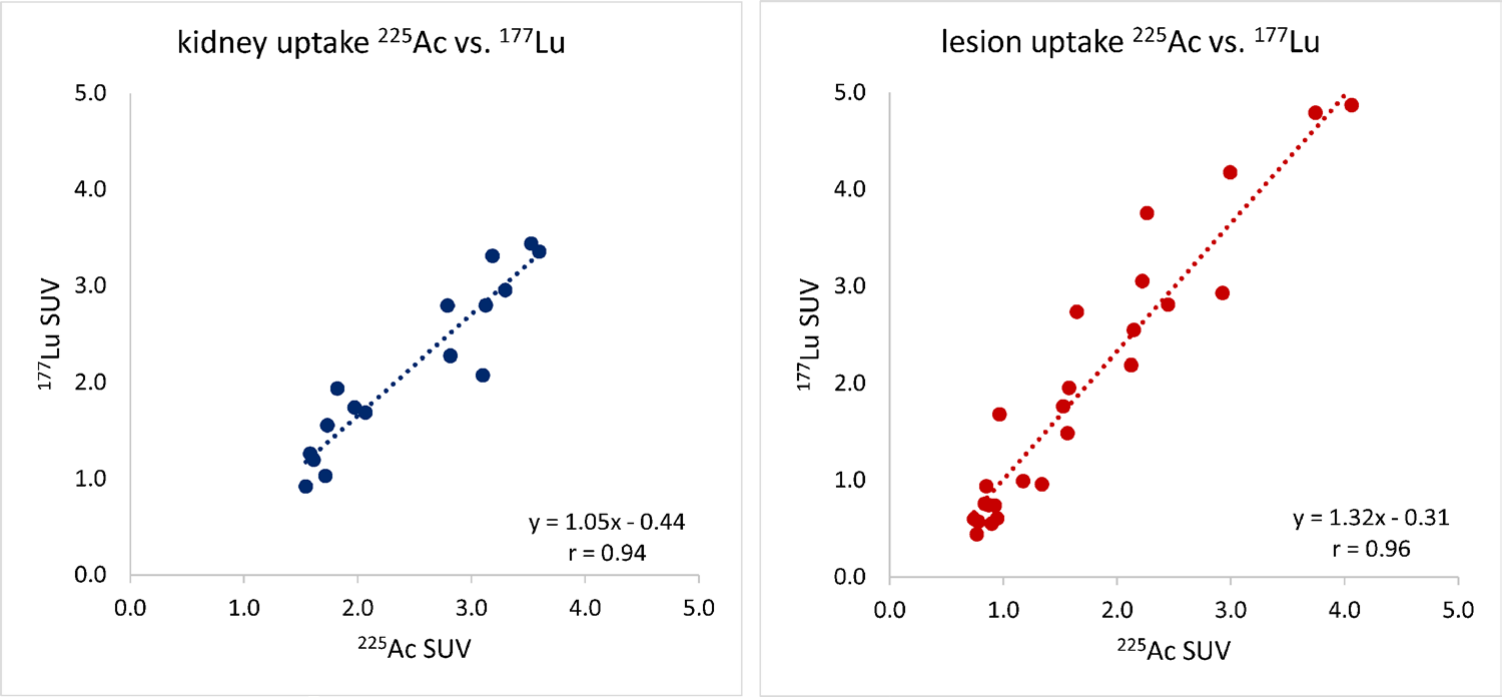
Comparison of kidney and lesion SUV for [^177^Lu]Lu-PSMA-I&T and [^225^Ac]Ac-PSMA-I&T at 24 h post injection

Figure 5 shows the SUV for [^177^Lu]Lu-PSMA-I&T and [^225^Ac]Ac-PSMA-I&T, respectively ^213^Bi, for all kidneys and 26 segmented lesions. For kidney and lesion SUV for [^177^Lu]Lu-PSMA-I&T and [^225^Ac]Ac-PSMA-I&T, a very strong and significant correlation was found (kidneys: Pearson’s *r* = 0.94, *p* < 0.01; lesions: Pearson’s *r* = 0.96, *p* < 0.01). The mean kidney SUV was 2.1 ± 0.9 and 2.5 ± 0.8 for [^177^Lu]Lu-PSMA-I&T and [^225^Ac]Ac-PSMA-I&T. Respective values for the not recovery-corrected lesion SUV were 2.1 ± 1.5 and 1.8 ± 1.1.

Statistical analysis via MATLAB Wilcoxon signed-rank testing revealed significant differences for the kidney SUV (*p* < 0.01) as well as lesion SUV (*p* = 0.03). The kidney SUV was found to be on average 21 ± 23% higher for [^225^Ac]Ac-PSMA-I&T, i.e., only lower compared to [^177^Lu]Lu-PSMA-I&T in one out of 16 kidneys. The tumor SUV was on average 7 ± 32% higher for [^177^Lu]Lu-PSMA-I&T. No recovery correction was considered in these data, neither for kidneys nor lesions. Recovery estimates for filtered ^177^Lu and ^225^Ac data in comparison can be found in the supplemental data. The average segmented lesion size was 21 ± 13 ml.

Dosimetry for patient 1 could not be evaluated, as only one image time point was available. Furthermore, seven lesions could not be evaluated via the mono-exponential fit model, as the maximum lesion uptake was detected at 48 h p. i., the remaining 15 lesions were available for dosimetry. The mono-exponential fit to the VOI data for [^177^Lu]Lu-PSMA-I&T, as collected at 24 h and 48 h p. i., revealed an effective half-life of 31 ± 17 h and 51 ± 39 h for the kidneys and lesions. The corresponding effective half-lives for [^225^Ac]Ac-PSMA-I&T were estimated as 33 ± 19 h and 61 ± 55 h under the assumption of equal biological compound pharmacokinetics for the ^225^Ac-labeled and ^177^Lu-labeled compound. Respective absorbed doses based on the harmonized and post-filtered data were estimated as 0.23 ± 0.13 Sv_RBE=1_/GBq and 0.20 ± 0.19 Sv_RBE=1_/GBq for kidneys and lesions for [^177^Lu]Lu-PSMA-I&T, respectively, and 0.28 ± 0.14 Sv_RBE=5_/MBq and 0.22 ± 0.21 Sv_RBE=5_/MBq for kidneys and lesions for [^225^Ac]Ac-PSMA-I&T. The kidney RBE-weighted absorbed dose for [^225^Ac]Ac-PSMA-I&T was higher for all investigated cases, resulting in an average increase of 32 ± 35% (*p* < 0.01). Regarding lesion dosimetry, no significant differences were found (*p* = 0.54). The RBE-weighted tumor absorbed dose was higher for [^225^Ac]Ac-PSMA-I&T in nine out of 15 lesions with an average increase of 21 ± 39%.

## Discussion

The HC phantom measurement demonstrates the general feasibility of SPECT imaging of the 440 keV photopeak of the ^225^Ac decay chain, i.e., imaging of the daughter radionuclide ^213^Bi. However, due to the high gamma energy, pronounced septal penetration is present during the Gamma camera measurement even with usage of a high-energy collimator. Chun et al. demonstrated for SPECT imaging of ^131^I that the application of conventional Gaussian-based resolution compensation models during SPECT reconstruction is limited in the case of dominant septal penetration [12]. By using more detailed and anisotropic models for the distance-dependent detector point-spread-function, SPECT imaging of high-energy gammas can be clearly improved. The employed reconstruction algorithm uses a stack of pre-simulated two-dimensional point-spread-functions for various distances.

For the LC measurement of the self-made phantom, i.e., the situation expected in the clinic, pronounced image noise in unfiltered data was visible. Thus, Gaussian post-filtering was necessary to derive a reasonable signal distribution. A FWHM of 30 mm was considered suitable, to allow for a trade-off between sufficient noise suppression on the one hand and an acceptable signal recovery on the other. However, a certain deformation of the insert signal still remained in the post-filtered images. The background recovery of less than 100% for both cases, HC and LC, indicates that the count yield coming from the background compartment (~ 900 Bq/ml) might already be too low to accurately restore the signal. Furthermore, the accuracy of conventional window-based scatter correction might be limited for the low-count situation, which should be considered in future investigations.

In agreement with the findings from the phantom study, a Gaussian post-filter with FWHM of 30 mm was applied to all patient data. This approach in general produced an uptake pattern which was in good agreement with the simultaneously acquired [^177^Lu]Lu-PSMA-I&T SPECT and the pre-therapy [^18^F]F-PSMA-1007 PET for all investigated patients. The strong post-filter seems to be justified considering the importance of noise suppression and the limited resolution of high-energy Gamma camera imaging. As it was the case for the LC phantom measurements, the activity distribution in post-filtered ^225^Ac SPECT patient data can be deformed or delocalized to a certain extent. The low number of acquired projections with approximately 11° angular step width in addition to the low resolution of ^225^Ac SPECT imaging makes it challenging to fully retrieve the signal distribution in highly complex uptake regions, e.g., bone lesions located in close proximity to the kidneys or the intestine. Ljungberg et al. proposed at least 128 views for a 128^3^-matrix [10]. However, the large step width used for ^225^Ac SPECT acquisitions resulted from the decision to favor a long measurement time per projection over the number of projections. SPECT algorithms that specifically address a sparse number of projections and extreme low-count situations should be further investigated for ^225^Ac SPECT imaging to improve image quality on the one hand and to reduce examination times on the other [31]. For therapeutic approaches with usage of the ^225^Ac compound alone, the simultaneous reconstruction of all gamma emissions could be employed to improve the count situation [16, 17]. However, accurate quantification would require that all photons originate from the same location. The latter might be complicated by the unknown mobility of potentially unbound daughter nuclides, which were released after the first alpha decay [32].

To allow for a spatial mapping between ^225^Ac and ^177^Lu SPECT imaging in particular for the lesions, Gaussian post-filtering was applied to the ^177^Lu-data as well to especially harmonize the SNR of kidneys and lesions for both nuclides under investigation. To account for the higher biological effect of alpha compared to beta radiation, an RBE of five was used for alpha dosimetry as proposed by Sgouros et al., although a range of RBE values might be conceivable [30]. Investigations in the scope of targeted alpha therapy, e.g., evaluation of therapy response, should also encompass to improve the knowledge on which RBE values should be applied to risk organs and lesions within the clinical setting.

Although the lesion SUV was significantly higher for [^177^Lu]Lu-PSMA-I&T at 24 h p. i., the prolonged effective half-life of ^225^Ac led to no significant differences regarding RBE-weighted lesion absorbed dose estimates. However, while ^225^Ac and ^177^Lu data were harmonized with regard to the image SNR, respective image recoveries still remained different, i.e., higher for ^177^Lu (see supplemental data). In this study, no recovery correction was applied to the lesion SUV or lesion absorbed doses. Recovery correction could be applied in principle; however, the true object recovery is dependent on multiple factors, such as object size, object-specific foreground-to-background ratio, and object shape, which is in general not known for a real patient case. Thus, phantom-based recovery factors can only provide an estimate for the expected quantification accuracy. The observed higher lesion SUV for [^177^Lu]Lu-PSMA-I&T of on average 7% might thus be partially driven by the nuclide-specific image recovery, despite no dependency of the uptake ratio with the lesion size was found.

The tumor pharmacokinetics was derived from two measurements only with the last imaging session being around 48 h post-therapy. Thus, the reported lesion absorbed doses represent only rough estimates. Feuerecker et al. reported bone lesion absorbed doses of 1.7 ± 1.13 Gy/GBq for [^177^Lu]Lu-PSMA-I&T therapy based on planar imaging until day seven post injection [33]. In the scope of the corresponding recovery estimates in harmonized data (supplemental data), the lesion absorbed doses reported for [^177^Lu]Lu-PSMA-I&T in this study are comparable to those of Feuerecker et al., considering the different imaging techniques used, i.e., planar imaging vs. SPECT, different patient cohorts, and potential differences regarding the applied tumor density. Absorbed doses are inversely proportional to the tissue density and in particular for bone lesion dosimetry, the respective lesion density can be significantly larger than one [29]. For [^177^Lu]Lu-PSMA-I&T, the found SPECT-based tumor-to-kidney ratios 24 h p. i. were similar to results from biodistribution analysis performed by Yusufi et al. [34]. Furthermore, ^177^Lu quantification using the high-energy collimator might be impaired by an inferior spatial resolution compared to the usage of a medium-energy collimator [10]. In this study, usage of the high-energy collimator aimed at the reduction of image-degrading septal penetration for imaging of the 440 keV photopeak.

The RBE-weighted kidney absorbed doses per administered activity were found to be significantly higher for [^225^Ac]Ac-PSMA-I&T. An average increase of the RBE-weighted absorbed dose per administered activity of 32% was found for the kidneys, whereupon a maximum value of up to 106% was observed. This increase can be attributed to both, a significantly higher kidney SUV plus the longer effective half-life for [^225^Ac]Ac-PSMA-I&T. For the patient with the largest difference in kidney absorbed doses per unit administered activity also the highest SUV ratio was observed. However, even under consideration of the determined recovery estimates, final kidney absorbed dose estimates could still be considered uncritical for [^225^Ac]Ac-PSMA-I&T. Kratochwil et al. estimated RBE-weighted kidney absorbed doses of 0.74 Sv_RBE=5_/MBq for [^225^Ac]Ac-PSMA-617 therapy by extrapolating existing pharmacokinetic data from [^177^Lu]Lu-PSMA-617 therapy and by assuming full local energy deposition of all daughter nuclides [35]. In this study, we combined the patient-specific [^177^Lu]Lu-PSMA-I&T pharmacokinetics with a single uptake measurement for [^225^Ac]Ac-PSMA-I&T and likewise assumed full local energy deposition for the ^225^Ac decay chain. Considering the limited kidney recovery coefficient in post-filtered data (supplemental data), the RBE-weighted kidney absorbed doses presented in this study are similar to those from Kratochwil et al., although patient cohorts differ and despite the use of different compounds. Furthermore, the assumption of full local energy deposition implies full internalization and intactness of the targeting molecule.

Within the proposed imaging protocol, the gamma emission originating from the ^213^Bi-decay was measured. The significantly higher kidney SUV could thus be attributed to both, a comparatively higher uptake of [^225^Ac]Ac-PSMA-I&T itself or the accumulation of potentially freely circulating ^213^Bi, that was released somewhere else in the patient body. Despite the applied post-filtering for harmonization of the image SNR, the image recovery can nonetheless be regarded as higher for ^177^Lu. Thus, it can be hypothesized that recovery correction would result in an even higher ^213^Bi uptake compared to [^177^Lu]Lu-PSMA-I&T (see supplemental data). The formation of free, unbound daughters during ^225^Ac-based radionuclide therapy is an ongoing topic of interest. The recoil energy released during the decay of ^225^Ac is sufficient to break up the chemical bonds of the targeting molecule [32]. Furthermore, the binding affinity of daughter nuclides to the chelator might be reduced due to different radiochemical properties [36]. Especially during the prolonged half-life of 45.6 min of ^213^Bi, a significant redistribution of unbound radionuclide can occur, unless sufficient internalization and thus trapping within the tumor cell can be ensured. Other attempts to prevent the circulation of unbound daughter nuclides focus on the encapsulation of ^225^Ac-labeled molecules in a specific carrier-system [37, 38]. Several studies reported the accumulation of freely circulating ^213^Bi in the kidneys [36, 39]. Schwartz et al. investigated the renal uptake and kidney dosimetry for radioimmunotherapy using ^225^Ac-huM195 based on biodistribution measurements in mice [39]. They found an effective half-life of 6.7 days for ^225^Ac and 4.6 days for so-called non-equilibrium, respectively freely circulating, ^213^Bi. For the dosimetry estimates derived in this study, we employed the biological wash-out determined from serial [^177^Lu]Lu-PSMA-I&T measurements and assumed that the kidney SUV, as it was measured for ^213^Bi, is equal to that for ^225^Ac. This could lead to an overestimation of the kidney absorbed dose, as part of the ^225^Ac decay chains might not have started in the kidneys but somewhere else in the patient body. Further investigations should focus on serial measurements of the time-dependent distribution of the different ^225^Ac progenies. As the alpha decays of the first two progenies ^221^Fr and ^217^At happen almost instantaneously, respective absorbed dose depositions could potentially be still PSMA-driven in a similar manner, while ^213^Bi might experience a different pharmacokinetic due to its potential mobility and the prolonged half-life. However, serial in vivo quantitative measurements would in particular require a further reduction of examination times. Furthermore, the current measurement time of 1 h per bed position severely limits the clinically usable field-of-view and poses a challenge for clinical capacities and patient compliance. Thus, further effort is required to pave the way towards a routine application of ^225^Ac imaging in the scope of targeted alpha therapy.

## Conclusion

The presented study demonstrates that quantitative SPECT imaging for [^225^Ac]Ac-PSMA-I&T therapy of metastasized prostate cancer is feasible despite extreme low-count conditions. Adequate post-filtering, i.e,. harmonization, of simultaneously acquired [^225^Ac]Ac-PSMA-I&T/[^177^Lu]Lu-PSMA-I&T SPECT data, led to visually similar uptake patterns, which were in agreement with pre-therapeutic [^18^F]F-PSMA-1007 PET/CT imaging. Kidney SUV at 24 h p. i. and RBE-weighted kidney absorbed doses were found to be significantly higher for [^225^Ac]Ac-PSMA-I&T compared to the ^177^Lu-based compound. The influences of the mobility of unbound daughter nuclides on quantitative imaging and dosimetry shall be further evaluated.

## Supplementary Information

Below is the link to the electronic supplementary material.

Supplementary file1 (DOCX 858 KB)

## Data Availability

Please contact the corresponding author.
