# Supplementary material for: Biodistribution and dosimetry for combined [177Lu]Lu-PSMA-I&T/[225Ac]Ac-PSMA-I&T therapy using multi-isotope quantitative SPECT imaging
Source: Eur J Nucl Med Mol Imaging. 2023 Jan 11;50(5):1280–90. doi: 10.1007/s00259-022-06092-1 (PMC10027798; doi:10.1007/s00259-022-06092-1)
Supplement: Supplementary file 1 — Supplementary file1 (DOCX 858 KB) [file 259_2022_6092_MOESM1_ESM.docx]

###### Supplement

**Simulation study**

In addition to the real phantom measurements performed for ^225^Ac only , SIMIND simulations (version 6.2.1) of a cylindrical phantom (3.5 l) containing three spherical inserts of varying size (200, 26.5, and 11.5 ml) were employed to directly compare quantitative imaging for ^177^Lu and ^225^Ac for the identical well known ground truth [[19](#_ENREF_19)]. The insert size was selected similar to a typical kidney volume and the two largest spheres of a conventional NEMA IEC body phantom. The foreground-to-background ratio was selected as 8:1 and the total phantom activity was 0.7 MBq for ^225^Ac and 88 MBq for ^177^Lu. While the real phantom measurement was performed for activity conditions at 72 h to determine the quantification protocol for the maximum expected time point according to the local conventional scanning protocol, the SIMIND simulation was set up for activity conditions around 24 h according to the imaging time point of ^177^Lu/^225^Ac SPECT/CT imaging used in this study. Thereby a ^225^Ac-to-^177^Lu activity ratio of 8:1000 was assumed according to the activity ratio administered during tandem therapy. The simulation parameters related to camera system and acquisition protocol as well as the reconstruction settings were identical to those employed for the real-life measurements. Image qualities for ^177^Lu and ^225^Ac are naturally different due to differences in spatial resolution and noise, which in particular impairs comparability of smaller structures. To harmonize respective image qualities varying Gaussian post-filters (20-50 mm full-width-half-maximum (FWHM) in steps of 5 mm) were applied to the ^177^Lu simulation. For ^225^Ac, a Gaussian post-filter with a FWHM of 30 mm was chosen in agreement with the pre-defined processing. Regarding the harmonization of ^177^Lu and ^225^Ac image qualities, a Gaussian post-filter with FWHM of 40 mm and 30 mm, respectively, provided the closest match of the signal-to-noise ratio of the two smallest inserts (Figure A1 and Table A1). Regarding the largest insert, which approximates a typical kidney volume, post-filters with 40 mm and 45 mm FWHM for ^177^Lu yielded comparable matches of the signal-to-noise ratio, however, 45 mm was chosen due to a slightly better match of the image recoveries. Simulation results are visualized in Fig. A2.


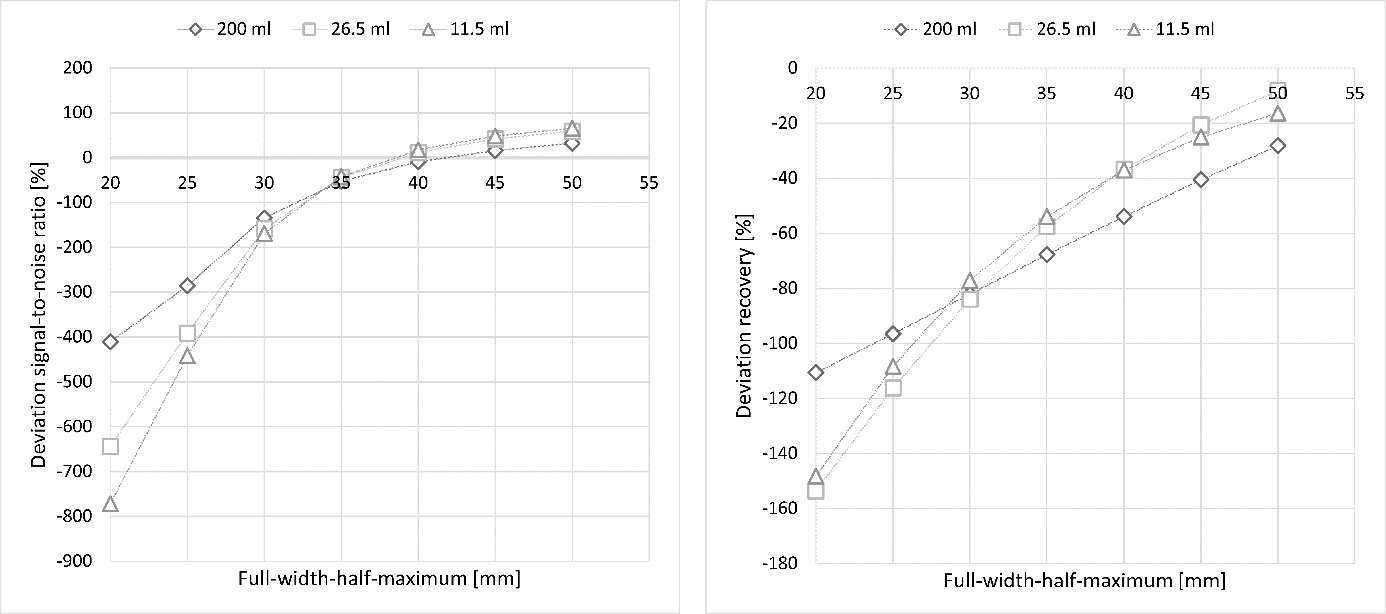


Fig. A1 Percentage deviation of ^177^Lu signal-to-noise ratio and image recovery depending on the applied Gaussian post-filter and in comparison to ^225^Ac (440 keV) imaging with a Gaussian post-filter of 30 mm full-width-half-maximum


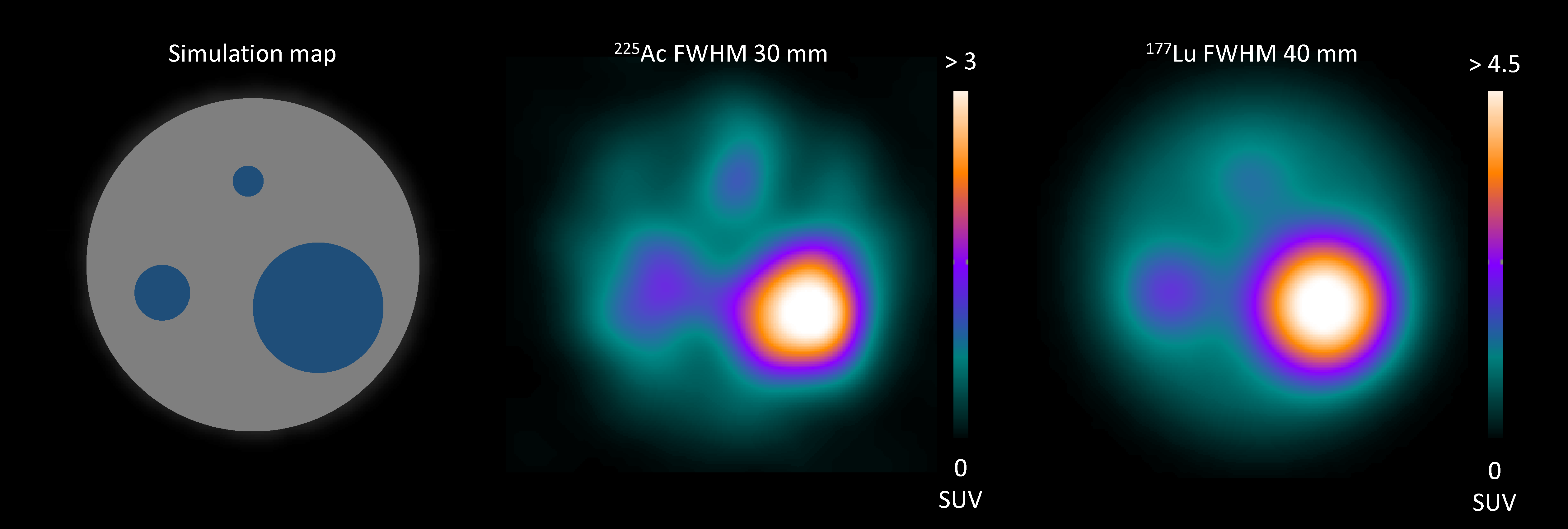


Fig. A2 Simulation results for the photopeak at 440 keV of ^225^Ac and the photopeak at 208 keV of ^177^Lu using post-filtering for harmonization of the nuclide-specific signal-to-noise ratio (Figure A1); respective image recoveries can be found in Table A1

Tab. A1 Recoveries (RC) and signal-to-noise ratio (SNR) for simulated sphere phantom filled with in total 0.7 MBq ^225^Ac and 88 MBq ^177^Lu at a foreground-to-background ratio of 8:1 (see Figure A2)

| Volume [ml] | RC [%]  ^177^Lu  no filter | RC [%]  ^177^Lu  45/40 mm FWHM | SNR  ^177^Lu  45/40 mm FWHM | RC [%]  ^225^Ac  30 mm FWHM | SNR  ^225^Ac  30 mm FWHM |
| --- | --- | --- | --- | --- | --- |
| 200 | 96 | 56/61 | 12/16 | 40 | 14 |
| 26.5 | 80 | 27/30 | 4/5 | 22 | 6 |
| 11.5 | 65 | 21/23 | 2/3 | 17 | 3 |

**Patient data**

**
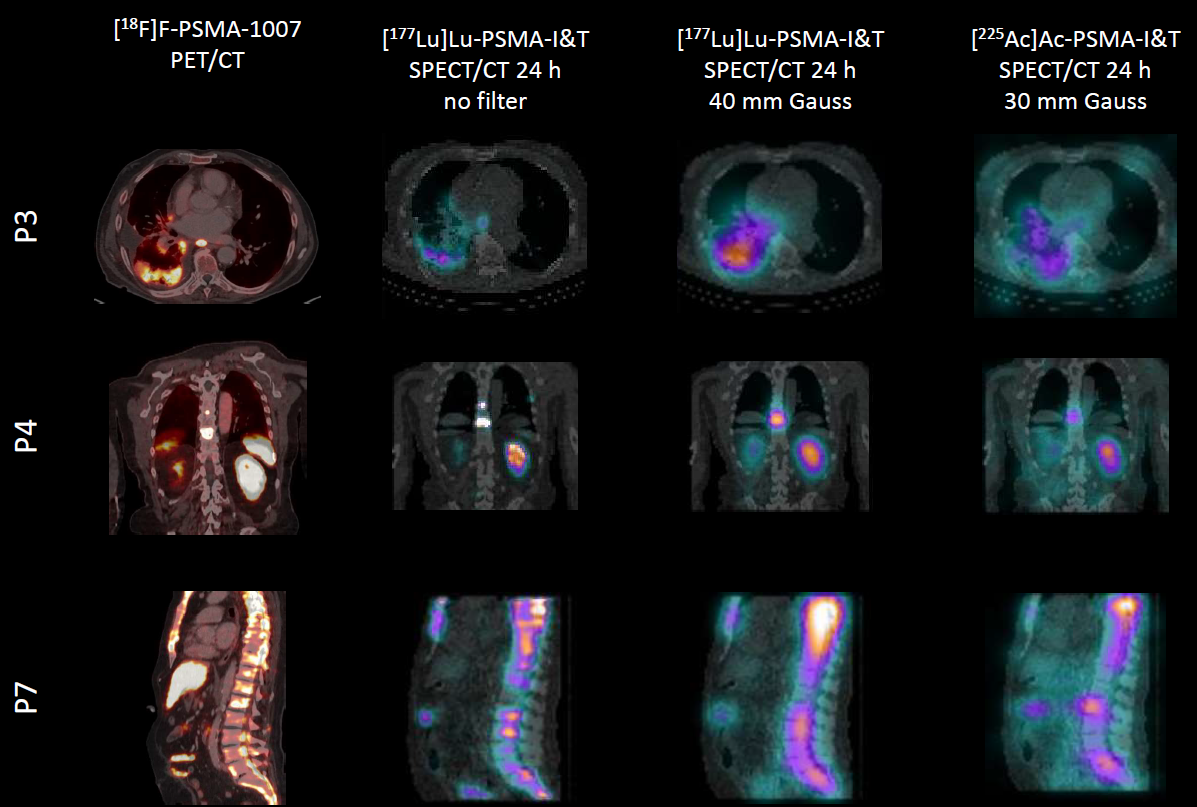
**

Fig. A3 Comparison of the pre-therapy PSMA PET/CT and post-therapy SPECT/CT 24 h after combined [^177^Lu]Lu-PSMA-I&T/[^225^Ac]Ac-PSMA-I&T therapy for patients P3, P4, P7; PET/CT and unfiltered ^177^Lu SPECT/CT images were scaled to the same maximum standardized uptake value, as well as post-filtered ^177^Lu and ^225^Ac SPECT/CT images

Tab. A2 Patient characteristics

| **Patient no.** | **1** | **2** | **3** | **4** | **5** | **6** | **7** | **8** |
| --- | --- | --- | --- | --- | --- | --- | --- | --- |
| Age [y] | 66 | 70 | 78 | 73 | 77 | 79 | 79 | 81 |
| Weight [kg] | 92 | 78 | 78 | 83 | 64 | 89 | 78 | 73 |
| PSA [ng/ml] | 594 | 189 | 37.3 | 40.4 | 1512 | 1111 | 147 | 692 |
| Lesions | OSS, LYM | OSS, LYM | OSS, LYM, PUL | OSS | OSS | OSS | OSS, LYM | OSS, LYM, PUL |
| Pre-therapies  (1: yes, 0: no) |  |  |  |  |  |  |  |  |
| - Surgery | 0 | 0 | 1 | 1 | 1 | 1 | 1 | 0 |
| - EBRT | 0 | 1 | 1 | 1 | 0 | 1 | 0 | 1 |
| - Anti-hormonal therapy (including Trenantone, Bicalutamide, Enzalutamide, Abiraterone) | 1 | 0 | 1 | 1 | 1 | 1 | 1 | 1 |
| - Chemotherapy (docetaxel, cabazitaxel) | 1 | 1 | 1 | 0 | 1 | 0 | 1 | 1 |
| - Olaparib | 1 | 0 | 0 | 0 | 1 | 0 | 0 | 0 |
| - [^223^Ra]RaCl_2_ | 1 | 0 | 0 | 1 | 0 | 1 | 0 | 1 |
| - [^177^Lu]Lu-PSMA-I&T | 1 | 1 | 1 | 1 | 1 | 1 | 0 | 1 |

metastases: OSS = osseous, LYM = lymph node, PUL=pulmonary; EBRT = external beam radiotherapy
